# lncRNA FLJ20021 regulates CDK1-mediated PANoptosis in a ZBP1-dependent manner to increase the sensitivity of laryngeal cancer-resistant cells to cisplatin

**DOI:** 10.1007/s12672-024-01134-6

**Published:** 2024-07-05

**Authors:** Xiaoyan Yin, Haizhong Zhang, Jingmiao Wang, Yanrui Bian, Qiaojing Jia, Zhichao Yang, Chunguang Shan

**Affiliations:** Department of Otolaryngology, The Second Hospital of Hebei Medical University, No. 215 Heping West Road, Xinhua District, Shijiazhuang, 050000 Hebei China

**Keywords:** Laryngeal cancer, lncRNA FLJ20021, CDK1, PANoptosis, Cisplatin resistance

## Abstract

In this study, we investigated the role of the newly discovered lncRNA FLJ20021 in laryngeal cancer (LC) and its resistance to cisplatin treatment. We initially observed elevated lncRNA FLJ20021 levels in cisplatin-resistant LC cells (Hep-2/R). To explore its function, we transfected lncRNA FLJ20021 and cyclin-dependent kinase 1 (CDK1) into Hep-2/R cells, assessing their impact on cisplatin sensitivity and PANoptosis. Silencing lncRNA FLJ20021 effectively reduced cisplatin resistance and induced PANoptosis in Hep-2/R cells. Mechanistically, lncRNA FLJ20021 primarily localized in the nucleus and interacted with CDK1 mRNA, thereby enhancing its transcriptional stability. CDK1, in turn, promoted panapoptosis in a ZBP1-dependent manner, which helped overcome cisplatin resistance in Hep-2/R cells. This study suggests that targeting lncRNA FLJ20021 can be a promising approach to combat cisplatin resistance in laryngeal cancer by regulating CDK1 and promoting PANoptosis via the ZBP1 pathway. These findings open up possibilities for lncRNA-based therapies in the context of laryngeal cancer.

## Introduction

Laryngeal cancer (LC) is the second most common type of head and neck squamous cell carcinoma (HNSCC) and is linked to a variety of factors, such as tobacco and alcohol use. According to statistics, there are 211,000 new cases and 126,000 deaths in the world every year [1, 2], which brings great health and economic burden to people. Even with current strategies, the overall 5-year survival rate for laryngeal cancer patients is only 64.2% [3]. In addition, more than 60% of laryngeal cancer patients have already been diagnosed with advanced throat cancer when they first receive their diagnosis [4]. Chemotherapy is the primary treatment method for patients with advanced and postoperative recurrent laryngeal cancer, and cisplatin (CDDP) is one of the most commonly used chemotherapy drugs, which serves on a toxic agent in carcinoma cells by destroying DNA and inducing cell death [5]. However, drug resistance in cancer limits the lethal effect of cisplatin on tumors [4, 6]. Therefore, this study aims to explore the new mechanism of cisplatin resistance from the perspective of cell death, with a view to developing more effective therapies.

PANoptosis is a new type of inflammatory cell death proposed by Malireddi et al. in 2019. It is characterized by simultaneous pyrodeath, apoptosis and necrotic apoptosis, but none of them can be explained by themselves [7]. At the same time, this mode of inflammatory cell death has been fully validated in various disease models, such as tumors and systemic sepsis [8, 9]. Recent studies have found that the progression of cancer and the development of chemical resistance problems are closely related to PANoptosis. For example, sulfaconazole induces PANoptosis by triggering oxidative stress and inhibiting glycolysis, thereby increasing the radiosensitivity of esophageal cancer [10]. Lin et al. found that phosphorylated NFS1 attenuates chemotherapy sensitivity to oxaliplatin in colorectal cancer by preventing PANoptosis [11]. Therefore, the emergence of PANoptosis fills the gap in cell death and may be a key breakthrough point in addressing cisplatin resistance in laryngeal cancer.

Long noncoding RNAs (lncRNAs), often defined as transcripts more than 200 nucleotides in length, can regulate the onset and progression of cancer [12]. In laryngeal cancer, several lncRNAs, such as ZFAS1 [13], AFAP1-AS1 [14], and NEAT1 [15], have been shown to be key players in malignant progression. In addition, abnormally expressed lncRNAs have been shown to be associated with increased chemical resistance to cancer cisplatin and may serve as promising therapeutic biomarkers for the treatment of laryngeal cancer [16, 17]. Therefore, identifying effective biomarkers and clarifying their regulatory mechanisms are of great significance. In a previous study, we used biogenic analysis to intersect chemotherapy resistance-related genes with differential genes in PANopsis and found that there were two coexpressed differential genes, namely, lncRNA FLJ20021 and KCNQ1OT1. Among them, lncRNA FLJ20021 is a newly discovered lncRNA whose key role and regulatory mechanism in laryngeal cancer resistance remain unclear.

Through bioanalysis of survival-related genes in laryngeal cancer patients, we discovered that CDK1 expression is related to the survival rate of laryngeal cancer patients and is probably a therapeutic target for laryngeal cancer. CDK1 is an important factor in the cell cycle control system, and increased CDK1 activity is common in tumor cells [18]. Recently, some bioinformatics studies have identified CDK1 as a pivotal gene associated with laryngeal squamous cell carcinoma, and the CDK1 gene is closely associated with malignant progression and poor outcome of tumors [19–21]. In addition, CDK1 regulates the pyrodeath, apoptosis and necrosis of adrenal cortical cancer cells (PANoptosis) by binding to PANoptosomes in a ZBP1-dependent manner [22]. Therefore, we speculate that CDK1 may be a key regulatory target that mediates PANoptosis and participates in cisplatin resistance in laryngeal cancer. However, the regulatory mechanisms and key roles of CDK1 and lncRNA FLJ20021 in laryngeal cancer need to be further elucidated.

Based on the above background, this study aims to investigate whether lncRNA FLJ20021 can affect cisplatin resistance in laryngeal cancer through PANoptosis and to explore how lncRNA FLJ20021 can participate in the regulation of cisplatin resistance in LC through the regulation of CDK1-mediated PANoptosis to provide promising therapeutic targets for the treatment of laryngeal cancer.

## Methods

### Cell culture and construction of cisplatin-resistant cells

Hep-2 and NP69 cells were purchased from American Type Culture Collection (ATCC) (Manassas, VA, USA). Cell cultures were performed according to the instructions provided by the ATCC. To construct cisplatin-resistant laryngeal cancer cells, Hep-2 cells were exposed to long-term culture in medium containing cisplatin (Aladdin, Shanghai, China) with gradually increasing concentrations of cisplatin (0, 1, 2, 4 and 8 μmol/L). At each cisplatin concentration, the cells were maintained for 3 months. We named the cisplatin-resistant cells Hep-2/R. The IC_50_ value of cisplatin in Hep-2 cells was used to determine the resistance of Hep-2 cells.

### RNA interference and plasmid transfection

Hep-2/R cells in the logarithmic growth stage were selected for grouping and experimental intervention and were divided into 6 groups: sh-NC group, sh-FLJ20021 group, pcDNA 3.1 group, pcDNA-CDK1 group, sh-FLJ20021 + pcDNA 3.1 group, and sh-FLJ20021 + pcDNA-CDK1 group. The short hairpin RNA and CDK1 overexpression plasmid vector targeting lncRNA FLJ20021 and their negative control were purchased from Jima Gene Co., Ltd. (Shanghai, China) and named sh-FLJ20021, sh-NC, pcDNA 3.1, and pcDNA-CDK1. These Rnas and plastids were transfected into Hep-2/R cells with Lipofectamine 3000 reagent (Invitrogen, Carlsbad, CA, USA).

### The IC50 of DDP determined by MTT assay

Hep-2/R cells at a density of 4 × 10^3^ cells/ml were inoculated on a 96-well plate and treated with cisplatin for 24 h. After 24 h, 10 μL MTT (0.5 mg/ml, Beyotime, Shanghai, China) was added to each well and incubated with aluminum foil at 37 °C and 5% CO_2_ for 4 h. Subsequently, formaldehyde crystals dissolved in 100 μL DMSO were added to each well, and the optical density was measured at dual wavelengths of 490/570 nm by an RT-6000 enzyme-label analyzer. The inhibitory effect of the test agent on cell growth was expressed as the maximum half (50%) inhibitory concentration (IC_50_).

### Colony formation experiment

The transfected Hep-2/R cells were inoculated in a 6-well plate at 1 × 103 cells per well. After 2 weeks, the cells were fixed with 4% paraformaldehyde (Beyotime, Shanghai, China) for 20 min and then stained with 0.2% crystal violet (Sigma‒Aldrich, St. Louis, MO, USA) for 5 min. The cells were then washed, and the number of colonies was counted under an inverted microscope. Clonal formation rate = number of clones/number of inoculated cells *100%.

### Flow cytometry

Hep-2/R cells were harvested, washed 3 times with PBS and incubated in the dark at room temperature with 5 μL Annexin V and PI (Beyotime, Shanghai, China) for 15 min. The apoptosis rate was detected by flow cytometry.

### YO-PRO-1/PI staining

The Dead cell assay kit (Beyotime, Shanghai, China) stained with YO-PRO-1 (YP1) and propyl iodide (PI) was used to detect apoptosis, necrosis, pyrodeath, etc., according to the specification. In short, Hep-2/R cells were cultured on a 24-well plate and treated with YP1 and PI reagents. Finally, the intensity of green and red fluorescence was observed under a fluorescence microscope.

### ELISA

After the specified treatment, the Hep-2/R cell supernatant was collected, and the concentrations of tumor necrosis factor-α (TNF-α) and interferon-γ (IFN-γ) were detected using a commercial ELISA kit (hnybio, Shanghai, China) according to the specification.

### Fluorescence in situ hybridization analysis (FISH)

The Hep-2/R cells were inoculated in sterile slides and fixed with 4% paraformaldehyde for 20 min. The sample was then permeated with 0.1% Triton X-100 (Biosharp, Shanghai, China) for 10 min and sealed at room temperature with 30 μL of prehybridization solution for 30 min. Subsequently, after washing with PBS buffer, the sample was incubated with fluorescent secondary antibody and lncRNA FLJ20021 FISH probe (Boxin Biotechnology Co., LTD., Guangzhou, China) at room temperature for 1 h. Next, the cells were rinsed with PBS buffer and incubated with DAPI staining solution at room temperature for 10 min. Finally, the images were visualized with a confocal laser scanning microscope.

### RNA immunoprecipitation (RIP)

The association of lncRNA FLJ20021 with CDK1 was determined using the Magna RIP RNA binding protein immunoprecipitation kit (Boxin Biotechnology Co., LTD., Guangzhou, China). In short, Hep-2/R cells were lysed with 100 μL of cell lysate, and the supernatant was collected by centrifugation at 4 °C. Subsequently, magnetic beads (Invitrogen, Waltham, MA, USA) were preincubated with Ago2 antibodies or IgG antibodies at room temperature for 1 h, followed by immunoprecipitation of the lysates using magnetic beads and overnight in a shaker at 4 °C. The next day, the RNA was purified from the RNA‒protein complex bound to the beads, and then the RNA levels were determined using RT‒qPCR.

### Quantitative real-time PCR (RT‒qPCR)

Total RNA was extracted from cells using TRIzol (Vazyme, Nnajing, China). cDNA was synthesized using a HiScript II first-strand cDNA synthesis kit (Vazyme, Nnajing, China). Gene expression was then detected by real-time PCR analysis of SIRT3 on a real-time fluorescent quantitative PCR apparatus (CFX96 Touch 1855195). Beta-actin and U6 served as internal controls. All primers used in this study are listed in Table 1. Gene expression was normalized using the 2^−∆∆Ct^ method.

**Table 1:** Primer sequences

Genes	Primer sequence (5ʹ-3ʹ)
lncRNA FLJ20021	F: 5ʹ-GAACCGAGGAGACGGGAGAA-3ʹ
	R: 5ʹ-AGATGGGTGTCAACTGTCCC-3ʹ
CDK1	F: 5ʹ-AGTCAGTCTTCAGGATGTGCTT-3ʹ
	R: 5ʹ-ATCCATGTACTGACCAGGAGGG-3ʹ
ZBP1	F: 5ʹ-GAATGCCAAGCACCCAAGAG-3ʹ
	R: 5ʹ-CCTTCAGGATCAGTCCCGC-3ʹ
GAPDH	F: 5ʹ-GGTCTCCTCTGACTTCAACA-3ʹ
	R: 5ʹ-GTGAGGGTCTCTCTCTTCCT-3ʹ

*F* Forward, *R* Reverse

### Western blotting assay

Proteins were extracted from cells and tissues using RIPA lysis buffer (Biosharp, Shanghai, China). Protein concentrations were determined by a BCA protein assay kit (NCM Biotech, Shanghai, China). All cell lysates containing 40 μg of protein were subjected to SDS‒PAGE and electrophoretically imprinted on PVDF membranes. The membrane was sealed with TTBS (Tween-Tris buffered brine) containing 5% skim milk at room temperature for 2 h and then incubated with the following primary antibodies: anti-CDK1 antibody (ab245318, Abcam, Cambridge, MA, USA), anti-GSDME antibody (ab209845, Abcam, Cambridge, MA, USA), anti-caspase-3 antibody (ab184787, Abcam, Cambridge, MA, USA), anti-caspase-7 antibody (ab255818, Abcam, Cambridge, MA, USA), anti-caspase-8 antibody (ab32125, Abcam, Cambridge, MA, USA), anti-MLKL (phospho S345) antibody (ab196436, Abclona, Wuhan, China), RIPK1-specific polyclonal antibody (175191AP, Abclona, Wuhan, China), phospho-RIPK1 (Ser161) monoclonal antibody (66854-1-Ig, Abclona, Wuhan, China), MLKL monoclonal antibody (66675-1-Ig, Abclona, Wuhan, China) (Proteintech, Rosemont, IL, USA), ZBP1 rabbit pAb (A13899, ABclonal, Wuhan, China), rabbit anti-caspase-1 antibody (bs-10442R, Servicebio, Beijing, China), rabbit anti-GSDMD antibody (bs-14287R, Servicebio, Beijing, China), and GAPDH-loading control (bsm-33033 M, Servicebio, Beijing, China). After that, the membrane containing protein bands was incubated with HRP polymerized secondary antibody (bs-0296G-HRP) at room temperature for 2 h. The bands were visualized by an ECL chemiluminescence detection system, and the protein expression was analyzed by ImageJ software for optical density values.

### Statistical analysis

Data were analyzed and plotted using GraphPad Prism 9 (Version 9.5.0, La Jolla, CA, USA). AI was used to collate the graph. All plots were represented by mean ± SD, and the significant difference between groups was tested by one-way test. A P-value less than 0.05 was considered a significant difference (* meant P < 0.05, ** meant P < 0.01, *** meant P < 0.001).

## Results

### FLJ20021 was highly expressed in LC and correlated with cisplatin resistance

**Fig. 1:**
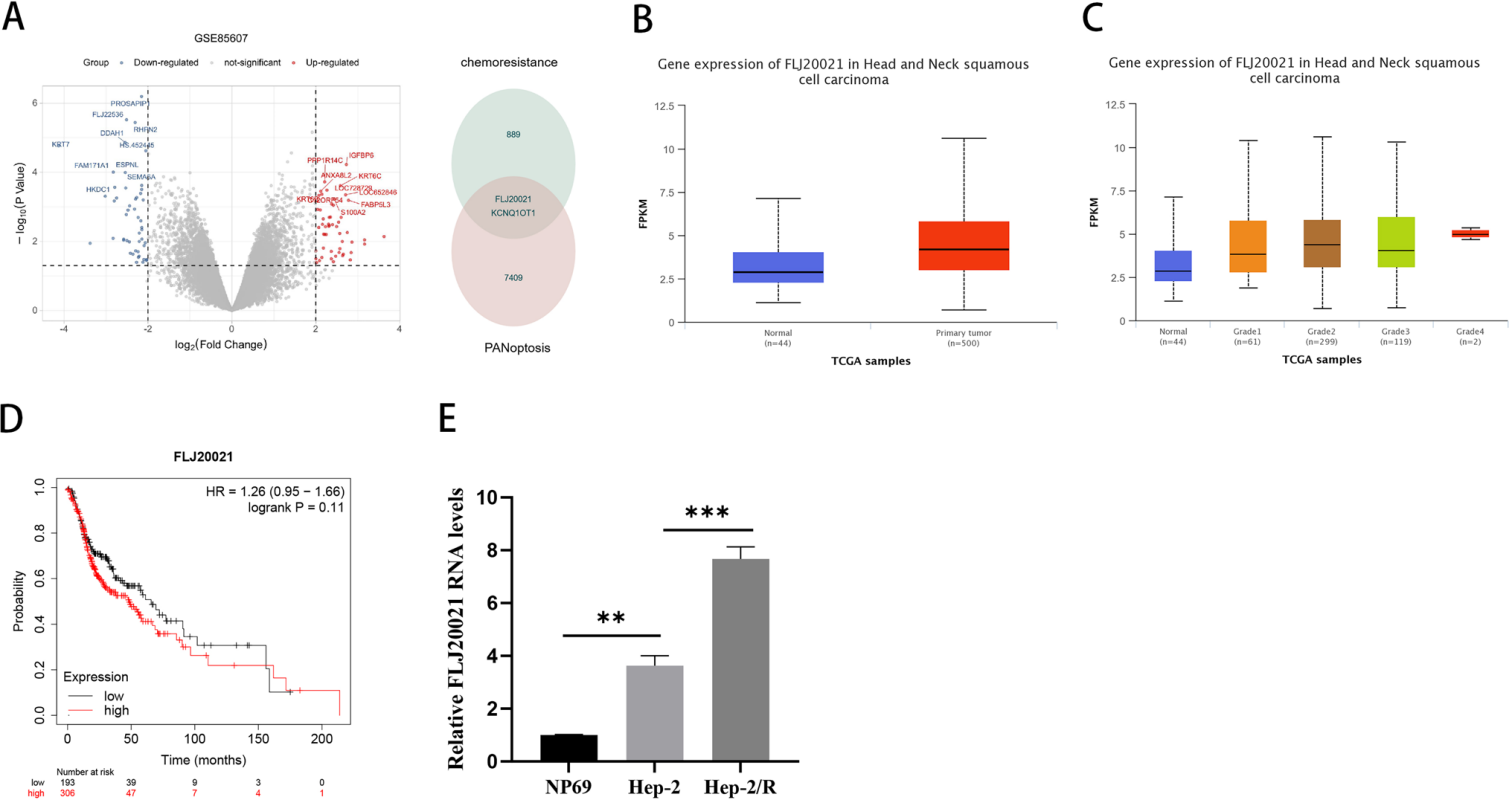
lncRNA FLJ20021 is highly expressed in laryngeal cancer and is associated with cisplatin resistance. **A** Volcano map of differentially expressed genes between chemically resistant samples in the GEO database and paracancerous tissues and intersection of differentially expressed genes with PANOS; **B** Expression of lncRNA FLJ20021 mRNA in primary HNSCC tissue and matched normal tissue; **C** Expression of lncRNA FLJ20021 mRNA in primary HNSCC tissues of patients with different clinical tumor grades from the UALCAN website server; **D** The OS value of HNSCC patients in different lncRNA FLJ20021 expression subgroups was analyzed by Kaplan‒Meier Plotter; **E** The different expression levels of FLJ20021 in NP69, Hep-2 and Hep-2/R cells were detected by qRT‒PCR assays; **: P < 0.01, ***: P < 0.001

In a previous study, data sets related to chemotherapy resistance were screened from the GEO database. A total of 889 differentially expressed genes (433 upregulated, 456 downregulated, P < 0.05 and |log2FC|> 1) were found by DESEq2 package analysis of R. Further intersection with the differential gene set of PANopsis revealed two coexpressed differential genes, namely, FLJ20021 and KCNQ1OT1 (Fig. 1A). However, the role and mechanism of FLJ20021 in LC resistance remain unclear. To determine the expression pattern of FLJ20021 in LC, we first analyzed the expression of FLJ20021 in head and neck cancer (HNSCC) and normal tissues using The Cancer Genome Atlas (TCGA) data sets. In the TCGA data, FLJ20021 mRNA levels were significantly elevated in HNSCC vs. normal tissues (Fig. 1B). The results of the public data analysis demonstrated that FLJ20021 expression was clearly visible between clinical grades and was significantly higher in patients with higher grades (Fig. 1C). Kaplan‒Meier Plotter Pan-Cancer RNA sequencing public web server (http://kmplot.com/analysis/index.php?p=service&cancer=pancancer_rnaseq) analysis results demonstrated that in all cases of HNSCC, higher levels of FLJ20021 were significantly associated with poorer overall survival (OS) (Fig. 1D). To determine the relationship between FLJ20021 and LC cisplatin resistance, we constructed LC cisplatin-resistant cells and named them Hep-2/R cells. Subsequently, RT‒qPCR detected the expression difference of FLJ20021 in NP69, Hep-2 and Hep-2/R cells. As shown in Fig. 1E, the mRNA level of FLJ20021 was elevated in Hep-2 and Hep-2/R vs. NP69 cells, and the expression level of FLJ20021 was the highest in Hep-2/R cells (P < 0.01).

In summary, FLJ20021 is upregulated in LC and may confer chemical resistance to laryngeal cancer.

### lncRNA FLJ20021 regulates CDK1-mediated PANoptosis to affect malignant progression and cisplatin resistance of LC cells

**Fig. 2:**
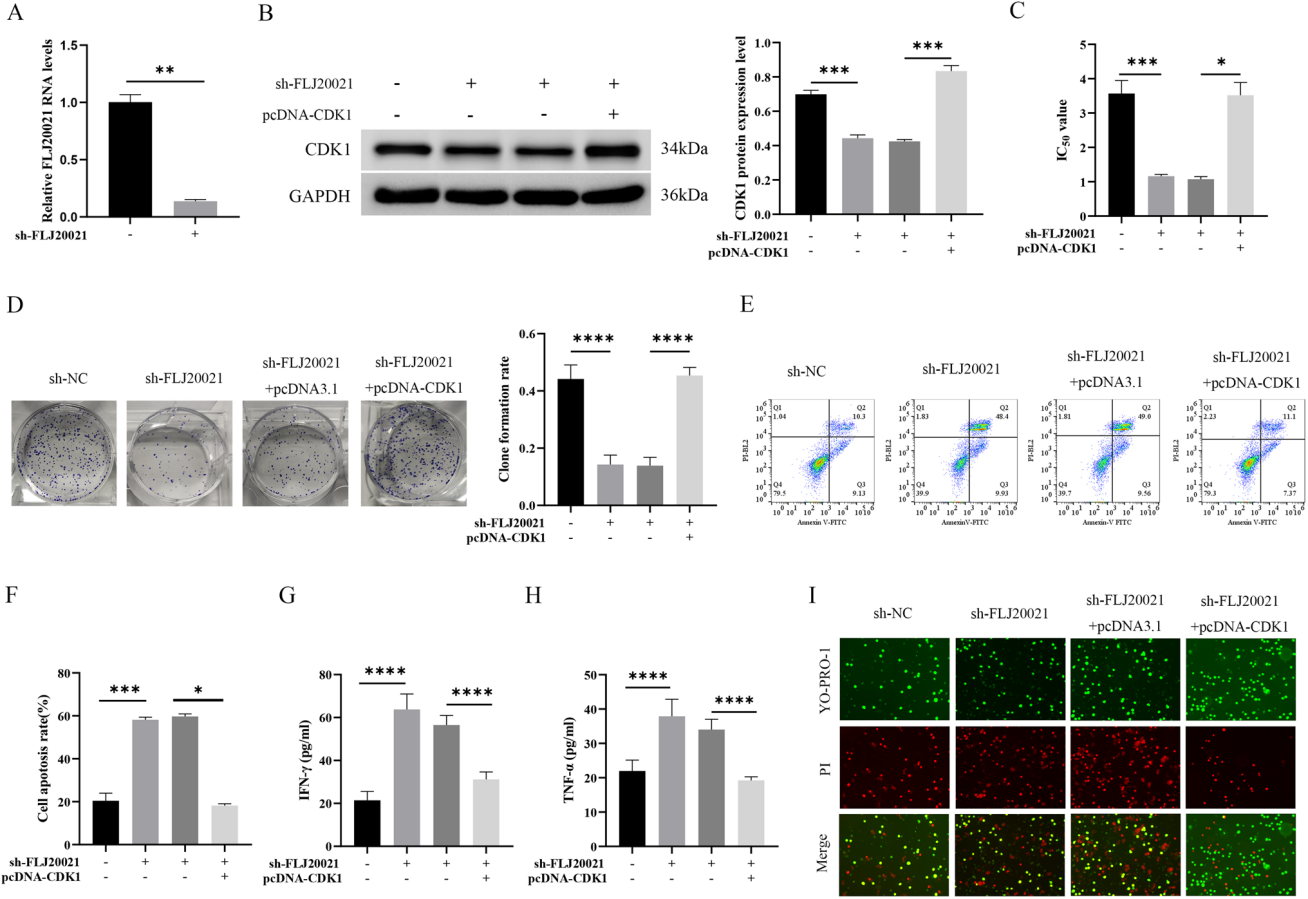
Effect of silencing lncRNA FLJ20021-mediated PANoptosis on cisplatin resistance in LC cells. sh-FLJ20021, sh-NC, sh-FLJ20021 and pcDNA 3.1 or pcDNA-CDK1 were transfected or cotransfected into Hep-2/R cells. **A** RT‒qPCR was used to detect the transfection efficiency of sh-FLJ20021 in cells; **B** Western blotting was used to detect the expression of CDK1 in cells. **C** The IC_50_ value of cells was detected by the MTT method; **D** Proliferation of cells was tested by the clonal formation assay; **E**/**F** Flow cytometry was applied to detect the apoptosis of cells; **G**/**H** The production of IFN-γ and TNF-α in the cell supernatant was detected by ELISA; **I** Apoptosis, necrosis, pyrodeath and iron death were detected by YO-PRO-1 (YP1) and propyridine iodide (PI) stained dead cell detection kit; *: P < 0.05, **: P < 0.01, ***: P < 0.001, ****: P < 0.0001

**Fig. 3:**
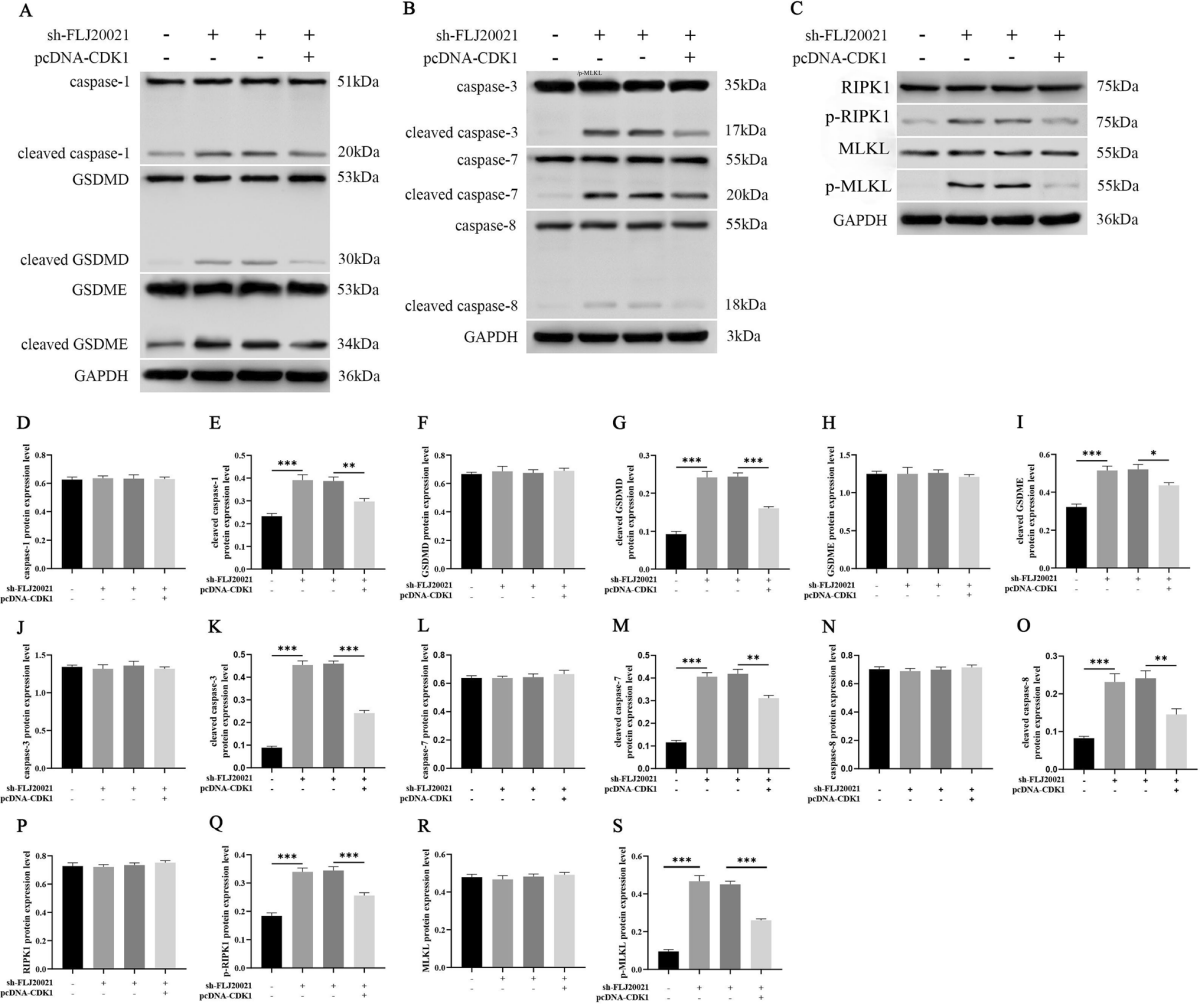
Silencing the effect of lncRNA FLJ20021 on key proteins of PANoptosis in cisplatin-resistant laryngeal carcinoma cells. **A**–**S** Western blot analysis of key proteins of PANoptosis, including pyro-related proteins (pro- and cleaved caspase-1, GSDMD/p-GSDMD and GSDME/p-GSDME), apoptosis-related proteins (pro- and cleaved caspase-3, pro- and cleaved) caspase-7, and pro- and cleaved caspase-8), and programmed necrosis (RIPK1/p-RIPK1, MLKL/p-MLKL); *: P < 0.05, **: P < 0.01, ***: P < 0.001

To investigate the functional role of lncRNA FLJ20021 in the malignant progression and cisplatin resistance of LC, a cell model of stable lncRNA FLJ20021 knockdown was first constructed. The transfection efficiency of sh-FLJ20021 was verified by RT‒qPCR. As shown in Fig. 2A, sh-FLJ20021 significantly depressed lncRNA FLJ20021 expression in Hep-2/R cells. Subsequently, sh-FLJ20021 and pcDNA 3.1 or pcDNA-CDK1 were transfected into Hep-2/R cells to determine whether CDK1 was involved in lncRNA FLJ20021-mediated PANoptosis and affected the malignant progression of laryngeal cancer and cisplatin resistance. The transfection efficiency was demonstrated by Western blotting, as shown in Fig. 2B. lncRNA FLJ20021 knockdown significantly decreased CDK1 expression in Hep-2/R cells, while pcDNA-CDK1 reversed this phenomenon. MTT assay results revealed that down-regulated lncRNA FLJ20021 depressed the IC_50_ value of Hep-2/R cells and enhanced cisplatin sensitivity, while pcDNA-CDK1 reversed this phenomenon (Fig. 2C). Clonal formation experiments revealed that knockdown of lncRNA FLJ20021 significantly lessened the clonal colony number of Hep-2/R cells, while overexpression of CDK1 reversed the inhibitory effect of sh-FLJ20021 on the proliferation of Hep-2/R cells (Fig. 2D). FCM revealed that lncRNA FLJ20021 knockdown significantly accelerated the apoptosis rate of Hep-2/R cells, whereas CDK1 overexpression reversed the promotion effect of sh-FLJ20021 on Hep-2/R cell proliferation (Fig. 2E, F). The effect of lncRNA FLJ20021 on PANoptosis was examined, and knockdown of lncRNA FLJ20021 combined with cisplatin treatment boosted cell death, including YP1-positive cells indicating apoptosis or necrosis and P1-positive cells indicating cell necrosis or pyrosis. However, overexpression of CDK1 reversed the effect of sh-FLJ20021 on PANoptosis (Fig. 2I). Studies have shown that tumor sensitivity to cell death and PANoptosis are related to TNF-α and IFN-γ expression profiles [23]. Therefore, the production of IFN-γ and TNF-α in the cell supernatant was detected by ELISA. As demonstrated in Fig. 2G and H, lncRNA FLJ20021 knockdown significantly boosted the concentrations of IFN-γ and TNF-α in the supernatant of Hep-2/R cells. However, overexpression of CDK1 reversed the promoting effect of sh-FLJ20021 on IFN-γ and TNF-α production in Hep-2/R cell supernatant. Subsequently, key proteins of PANoptosis were analyzed by Western blotting. As demonstrated in Fig. 3A and Fig. 3D–I, caspase-1, GSDMD and GSDME were detected in Hep-2/R cells. lncRNA FLJ20021 knockdown further upregulated cleaved caspase-1 expression, and GSDMD and GSDME were strongly activated, indicating that pyroptosis occurred. In addition, proteolytic cleavage of apoptotic caspases, including upstream caspase-8 and downstream caspase-3 and -7, was observed in lncRNA FLJ20021 knockdown-treated Hep-2/R cells, suggesting sensitivity to lncRNA FLJ20021 knockdown-induced apoptotic effector activation (Fig. 3B, J–O). In addition, elevated levels of phosphorylated RIPK1 and phosphorylated MLKL were observed in lncRNA FLJ20021 knockdown-treated Hep-2/R cells, indicating possible programmed necrosis (Fig. 3C, P–S). This suggests that lncRNA FLJ20021 suppresses PANoptosis in LC cells treated with cisplatin, including simultaneous activation of apoptosis, necrosis, and pyrosis. However, overexpression of CDK1 reversed the effect of sh-FLJ20021 on PANoptosis (Fig. 3A–S).

These data manifest that lncRNA FLJ20021 may mediate the effects of CDK1-mediated PANoptosis on cisplatin resistance in LC.

### CDK1 in LC is regulated by lncRNA FLJ20021 transcription

**Fig. 4:**
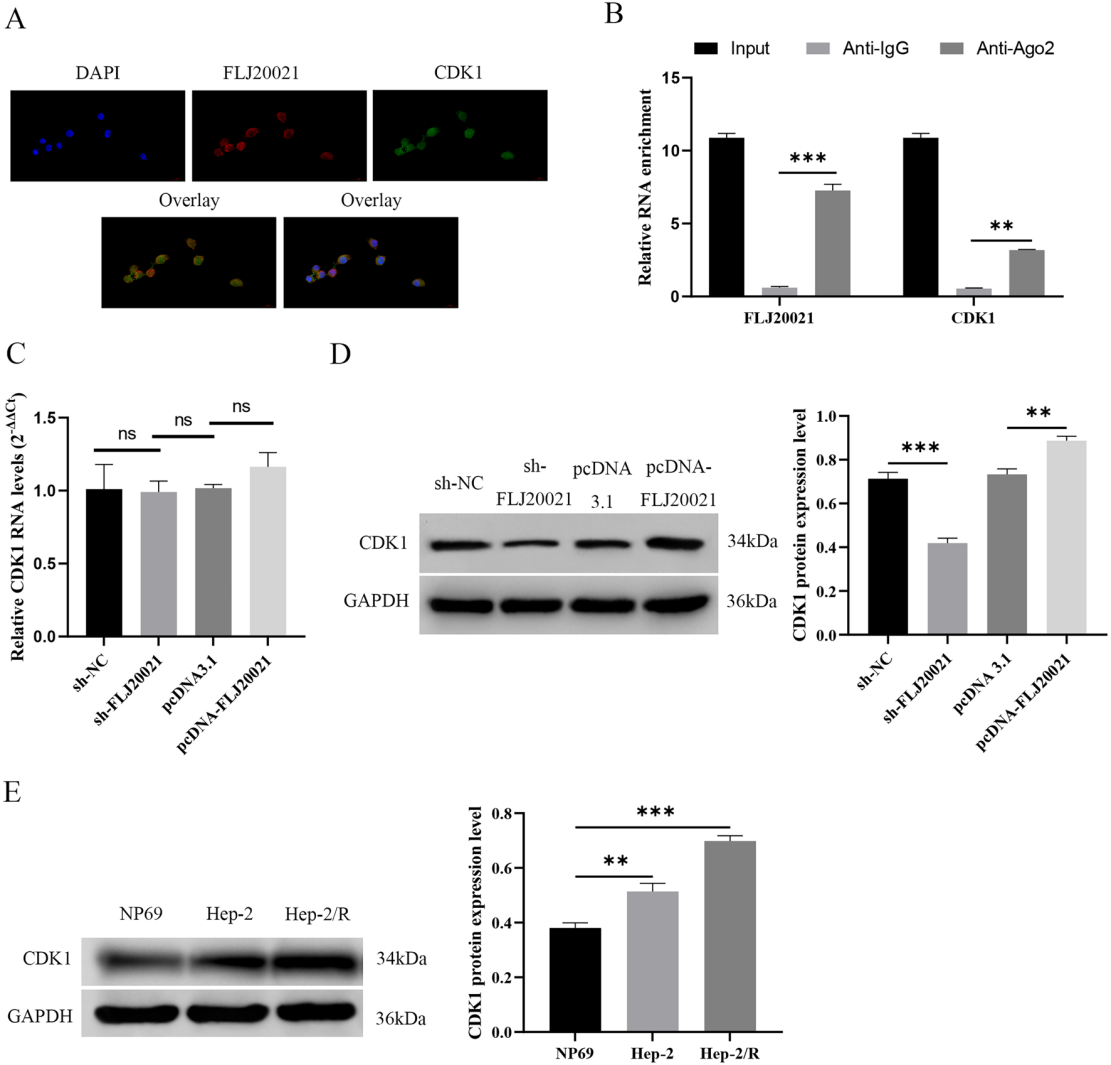
CDK1 is adjusted by lncRNA FLJ20021 transcription in LC. **A** FISH analysis evaluated the distribution of lncRNA FLJ20021 and CDK1 in the cytoplasm and nucleus of Hep-2/R cells; **B** RIP assay was applied to analyze the interaction between lncRNA FLJ20021 and CDK1; **C**/**D** Effects of knockdown or overexpression of lncRNA FLJ20021 on CDK1 mRNA and protein expression in laryngeal cancer by RT‒qPCR and Western blot analysis; **E** The expression difference of CDK1 in NP69, Hep-2 and Hep-2/R were detected by Western blot; **: P < 0.01, ***: P < 0.001

To analyze the mechanism of lncRNA FLJ20021, the localization of lncRNA FLJ20021 in cells was first analyzed by FISH assay. As manifested in Fig. 4A, both lncRNA FLJ20021 and CDK1 were mainly distributed in the nucleus of Hep-2 cells, indicating that lncRNA FLJ20021 may regulate gene transcription and affect mRNA splicing, stability and translation. To further confirm the binding between lncRNA FLJ20021 and CDK1, we conducted RIP experiments. As manifested in Fig. 4B, both lncRNA FLJ20021 and CDK1 were abundant in the RNA-induced silencing complex of Ago2 antibody immunoprecipitation but not in the RNA-induced silencing complex of IgG antibody immunoprecipitation. Furthermore, we found that knockdown or overexpression of lncRNA FLJ20021 in Hep-2/R cells did not affect CDK1 mRNA expression (Fig. 4C) but depressed and boosted CDK1 protein expression, respectively (Fig. 4D). We also observed that CDK1 was upregulated in Hep-2 and Hep-2/R cells compared to NP69 cells, with the most significant increase in expression in Hep-2/R cells (P < 0.01) (Fig. 4E).

Together, this evidence suggests that CDK1 is highly expressed in LC and that lncRNA FLJ20021 may be participated in the regulation of cisplatin resistance in LC by interacting with CDK1 mRNA and modulating its transcriptional stability.

### CDK1 mediates the influence of PANoptosis on cisplatin resistance in LC in a ZBP1-dependent manner

**Fig. 5:**
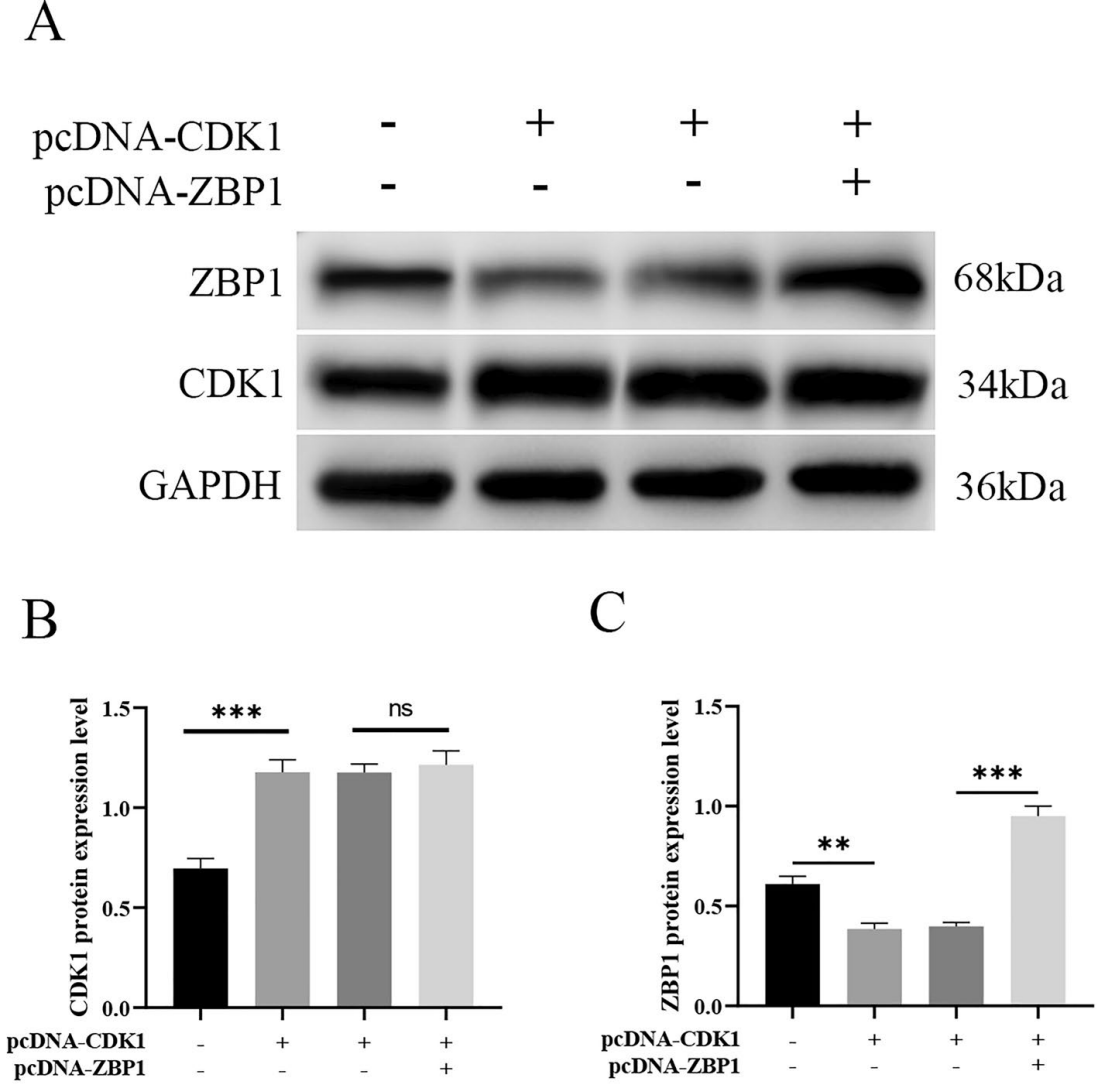
CDK1 upregulates ZBP1 expression in cisplatin-resistant laryngeal cancer cells. pcDNA 3.1, pcDNA-CDK1, pcDNA-CDK1 and pcDNA-3.1 or pcDNA-pcDNA-ZBP1 were transfected or cotransfected into Hep-2/R cells. **A**–**C** The transfection efficiency of the plasmid in Hep-2/R cells was determined by Western blot analysis; **: P < 0.01, ***: P < 0.001

**Fig. 6:**
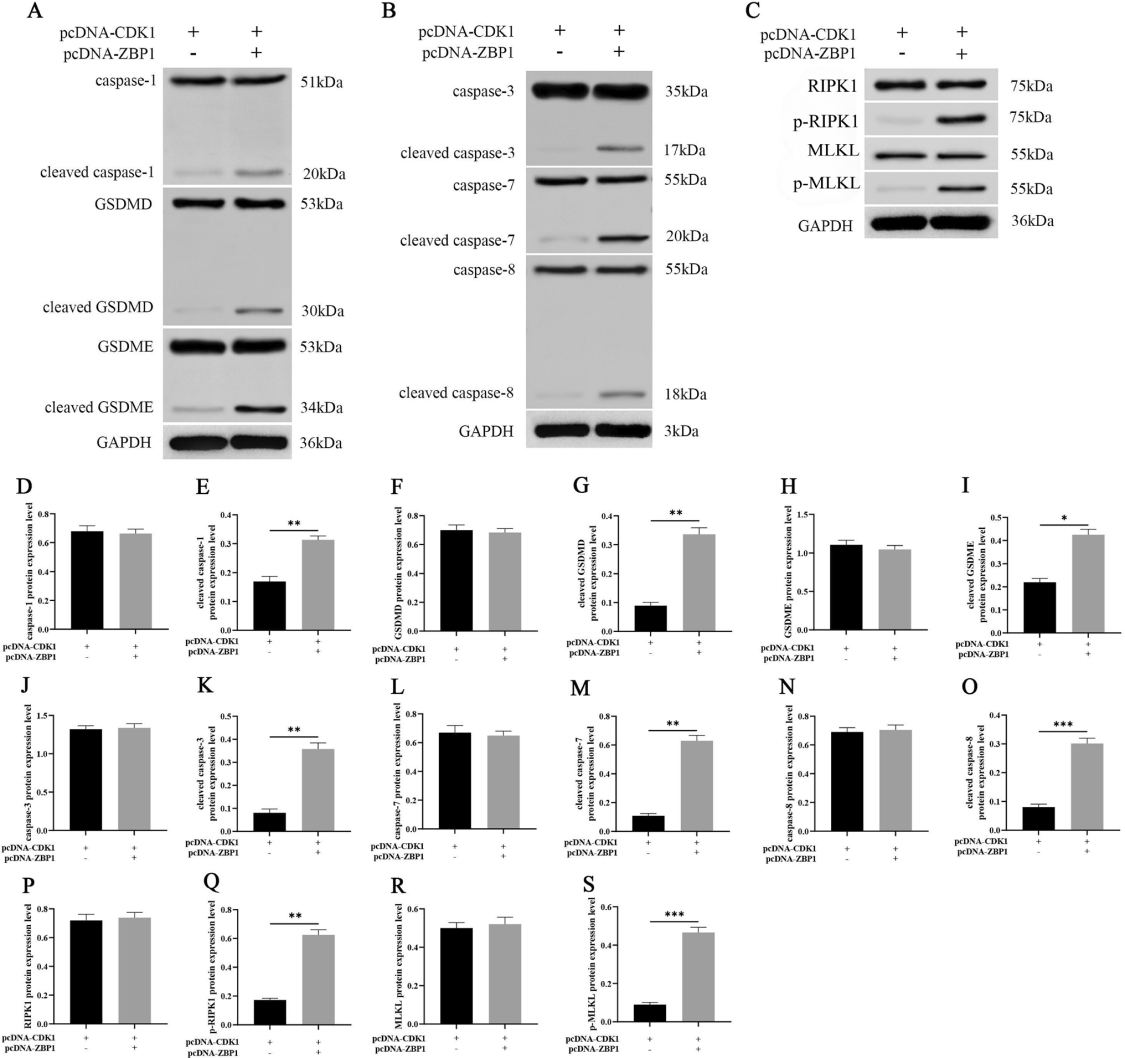
CDK1 mediates the influence of PANoptosis on cisplatin resistance in LC in a ZBP1-dependent manner. **A**–**S** Western blot analysis of key proteins of PANoptosis, including pyroptosis-related proteins (pro- and cleaved Caspase-1, GSDMD/p-GSDMD and GSDME/p-GSDME), apoptosis-related proteins (pro- and cleaved caspase-3, pro- and cleaved) caspase-7, and pro- and cleaved caspase-8), and programmed necrosis-related proteins (RIPK1/p-RIPK1, MLKL/pMLKL); *: P < 0.05, **: P < 0.01, ***: P < 0.001

Previous studies have revealed that overexpression of ZBP1 significantly promotes PANoptosis in cancer cells [24]. To further investigate whether CDK1 can mediate the influence of PANoptosis on cisplatin resistance in laryngeal cancer by regulating ZBP1, pcDNA 3.1, pcDNA-CDK1 and pcDNA 3.1 or pcDNA-pcDNA-ZBP1 were transfected or cotransfected into Hep-2/R cells. Western blot analysis showed that upregulation of CDK1 significantly downregulated ZBP1 expression in cells, while overexpression of ZBP1 reversed the upregulation of CDK1 on the expression of ZBP1 in Hep-2/R cells (Fig. 5A–C). Subsequently, key proteins of PANoptosis were analyzed by Western blotting. As shown in Fig. 6A–S, overexpression of ZBP1 rescued the effect of sh-CDK1 on key PANoptosis proteins. This indicates that CDK1 may mediate the influence of PANoptosis on cisplatin resistance in LC in a ZBP1-dependent manner.

## Discussion

In our study, we identified a novel lncRNA, lncRNA FLJ20021, as an important mediator of PANoptosis and cisplatin resistance promotion in laryngeal cancer. Higher lncRNA FLJ20021 expression was positively associated with poor prognosis of laryngeal cancer. We found that targeted inhibition of lncRNA FLJ20021 using RNA-infected plasmids in laryngeal cancer cells enhances the chemotherapy sensitivity of tumor cells by activating PANoptosis. Further mechanistic studies showed that upregulated lncRNA FLJ20021 can bind to CDK1, resulting in increased transcriptional activity of CDK1. In addition, we demonstrated that lncRNA FLJ20021 mediates the influence of PANoptosis on cisplatin resistance in LC in a ZBP1-dependent manner by regulating CDK1. Therefore, our results suggest the importance of lncRNA FLJ20021 as a novel therapeutic target for laryngeal cancer.

In recent years, according to a literature review, lncRNAs have become important regulators of various malignant tumors, including laryngeal cancer [25]. For example, DLX6-AS1 [26], NEAT1 [15], ZFAS1 [13] and NKILA [27] regulate the progression of laryngeal cancer through different regulatory mechanisms. Therefore, here, we used bioinformatics to screen lncRNAs for differential gene intersections of chemotherapeutic resistance and PANoptosis. Based on the analysis results, we chose lncRNA FLJ20021 as the focus of our study because it is significantly associated with chemotherapy resistance and PANoptosis. In addition, we first analyzed lncRNA FLJ20021 expression through the Shengxin website and found that lncRNA FLJ20021 was abnormally highly expressed in head and neck squamous cell carcinoma (HNSC), and patients with high levels of lncRNA FLJ20021 had worse long-term survival. Since most cancer cells can escape apoptosis, uncontrolled proliferation occurs [28]. In addition, they can become resistant to chemotherapy drugs through the programmed cell death pathway (PCD) [29]. Pyroptosis, apoptosis, and necrotic apoptosis are three key PCD pathways, and PANoptosis has features of pyroptosis, apoptosis, and necrotic apoptosis simultaneously [9, 30]. Therefore, understanding new programmed cell death pathways is particularly important to combat drug resistance. Next, we conducted a series of functional experiments to evaluate the effect of lncRNA FLJ20021-mediated PANoptosis on cisplatin resistance in laryngeal cancer. The results showed that inhibition of lncRNA FLJ20021 with targeted shRNA inhibited the proliferation and IC of cisplatin-resistant laryngeal cancer cells and promoted apoptosis. Further studies showed that lncRNA FLJ20021 inhibition of laryngeal cancer cells treated in concert with cisplatin induced powerful PANoptosis, including simultaneous activation of apoptosis, necrosis, and pyrodeath. This leads us to speculate that lncRNA FLJ20021 may be a broad target of cisplatin chemotherapy susceptibility and that lncRNA FLJ20021 deficiency may enhance antitumor effects by promoting the PANoptosis pathway.

CDK1 is an important emerging target in cancer that plays a key role in cell cycle progression through G2/M phase transitions and activation of homologous recombination (HR) DNA repair pathways [31]. CDK1 has been reported to play a crucial role in the malignant progression and chemical resistance of multiple cancers, including human pancreatic ductal adenocarcinoma [32], ovarian cancer [33] and colorectal cancer [34]. It is noteworthy that CDK1 can be involved in the regulation of PANoptosis in tumors [22]. In addition, we also note that some bioinformatics studies have identified CDK1 as a pivotal gene associated with laryngeal squamous cell carcinoma, and the CDK1 gene is strongly associated with malignant progression and poor outcome of tumors [19–21]. Therefore, we targeted CDK1 as a candidate downstream target gene of lncRNA FLJ20021 in laryngeal cancer. FISH experiments showed that lncRNA FLJ20021 and CDK1 are mainly distributed in the nucleus. Further detection by RIP, RT‒qPCR and Western blot analysis showed that lncRNA FLJ20021 was a positive regulator of CDK1. Next, we further explored whether CDK1 was involved in lncRNA FLJ20021's regulation of PANoptosis's influence on cisplatin resistance in LC. Functional rescue experiments showed that overexpression of CDK1 can reverse the effect of lncRNA FLJ20021 knockdown on the proliferation of cisplatin-resistant cells and PANoptosis in laryngeal cancer, suggesting that lncRNA FLJ20021 can mediate the influence of PANoptosis on cisplatin resistance in LC by regulating CDK1.

Recent studies have shown that ZBP1 is an upstream receptor of pyroptosis, apoptosis and necrosis and is involved in driving inflammatory cell death (PANoptosis) [35]. ZBP1 is an important innate immune sensor that adjusts inflammation during pathogen invasion [36]. We note that CDK1 regulates pyrodeath, apoptosis, and necrosis of cancer cells (PANoptosis) by binding to PANoptosomes in a ZBP1-dependent manner [22]. However, it is unclear whether CDK1 can induce PANoptosis by activating ZBP1 and further causing LC cells to develop. Therefore, to further explore whether CDK1 can mediate the effect of PANoptosis on the cisplatin resistance of LC by regulating ZBP1, we found that the expression of ZBP1 is downregulated after overexpression of CDK1 in LC cisplatin-resistant cells, and the overexpression of ZBP1 can rescue the influence of overexpression of CDK1 on key proteins of PANoptosis. This indicates that CDK1 mediates the effect of PANoptosis on cisplatin resistance in LC in a ZBP1-dependent manner.

Although our data suggest that lncRNA FLJ20021 is an oncogene in laryngeal cancer, there are still some limitations. First, this study is based on biogenic analysis and cell culture investigations and has not been studied in animals, let alone generalized to patients. Second, due to the variety of lncRNA targets, we cannot rule out the possibility that lncRNA FLJ20021 is involved in the regulation of other transcription factors and signaling pathways. Therefore, more investigations are needed in the future to further elucidate this issue.

## Conclusion

lncRNA FLJ20021 is highly expressed in cisplatin-resistant laryngeal cancer cells, and silencing lncRNA FLJ20021 plays a positive role in overcoming cisplatin-resistant laryngeal cancer by regulating CDK1 and mediating PANoptosis in a ZBP1-dependent manner.

lncRNA FLJ20021 may be a broad target for susceptibility to cisplatin chemotherapy, and lncRNA FLJ20021 deficiency may enhance anti-laryngeal cancer tumor effects. This study may provide a possible lncRNA-targeted therapy for laryngeal cancer.

## Data Availability

The data that support the findings of this study are available from the corresponding author upon reasonable request.
